# The Lumbar Stenosis Prognostic Subgroups for Personalizing Care and Treatment (PROSPECTS) study: protocol for an inception cohort study

**DOI:** 10.1186/s12891-022-05598-x

**Published:** 2022-07-21

**Authors:** Sean D. Rundell, Ayumi Saito, Eric N. Meier, Stephanie T. Danyluk, Jeffrey G. Jarvik, Kelley Seebeck, Janna L. Friedly, Patrick J. Heagerty, Sandra K. Johnston, Monica Smersh, Maggie E. Horn, Pradeep Suri, Amy M. Cizik, Adam P. Goode

**Affiliations:** 1 Department of Rehabilitation Medicine, University of Washington, Box 356490, 1959 NE Pacific St, Seattle, WA 98195-6490 USA; 2 Department of Health Systems and Population Health, University of Washington, Seattle, WA USA; 3 Clinical Learning, Evidence, and Research Center, University of Washington, Seattle, WA USA; 4 Department of Epidemiology, University of Washington, Seattle, WA USA; 5 Department of Biostatistics, University of Washington, Seattle, WA USA; 6 Department of Orthopaedic Surgery, Duke University, Durham, NC USA; 7 Department of Radiology, University of Washington, Seattle, WA USA; 8 Department of Neurological Surgery, University of Washington, Seattle, WA USA; 9 Department of Population Health Sciences, Duke University, Durham, NC USA; 10 Division of Rehabilitation Care Services, Veteran Affairs Puget Sound Health Care System, Seattle, WA USA; 11 Seattle Epidemiologic Research and Information Center, Veteran Affairs Puget Sound Health Care System,, Seattle, WA USA; 12 Department of Orthopaedics, University of Utah, Salt Lake City, UT USA; 13 Duke Clinical Research Institute, Duke University, Durham, NC USA

**Keywords:** Lumbar spinal stenosis, Spine pain, Cohort study, Prognostic model

## Abstract

**Background:**

Lumbar spinal stenosis (LSS) is a common degenerative condition that contributes to back and back-related leg pain in older adults. Most patients with symptomatic LSS initially receive non-operative care before surgical consultation. However, there is a scarcity of data regarding prognosis for patients seeking non-surgical care. The overall goal of this project is to develop and evaluate a clinically useful model to predict long-term physical function of patients initiating non-surgical care for symptomatic LSS.

**Methods:**

This is a protocol for an inception cohort study of adults 50 years and older who are initiating non-surgical care for symptomatic LSS in a secondary care setting. We plan to recruit up to 625 patients at two study sites. We exclude patients with prior lumbar spine surgeries or those who are planning on lumbar spine surgery. We also exclude patients with serious medical conditions that have back pain as a symptom or limit walking. We are using weekly, automated data pulls from the electronic health records to identify potential participants. We then contact patients by email and telephone within 21 days of a new visit to determine eligibility, obtain consent, and enroll participants. We collect data using telephone interviews, web-based surveys, and queries of electronic health records. Participants are followed for 12 months, with surveys completed at baseline, 3, 6, and 12 months. The primary outcome measure is the 8-item PROMIS Physical Function (PF) Short Form. We will identify distinct phenotypes using PROMIS PF scores at baseline and 3, 6, and 12 months using group-based trajectory modeling. We will develop and evaluate the performance of a multivariable prognostic model to predict 12-month physical function using the least absolute shrinkage and selection operator and will compare performance to other machine learning methods. Internal validation will be conducted using k-folds cross-validation.

**Discussion:**

This study will be one of the largest cohorts of individuals with symptomatic LSS initiating new episodes of non-surgical care. The successful completion of this project will produce a cross-validated prognostic model for LSS that can be used to tailor treatment approaches for patient care and clinical trials.

**Supplementary Information:**

The online version contains supplementary material available at 10.1186/s12891-022-05598-x.

## Background

Lumbar spinal stenosis (LSS) is a common degenerative condition that contributes to back and back-related leg pain in older adults [1]. The most common symptoms of LSS include leg and back pain that are typically worse with standing and walking and better with sitting or forward bending [1]. Imaging studies indicate that the prevalence of anatomic LSS is approximately 39% among those over age 60 [2]. However, while anatomic spinal stenosis is not always symptomatic, symptomatic LSS is still common, with an estimated prevalence of 11% in the general population [3–5]. Individuals with LSS have greater disability and are at a higher risk of persistent back-related disability compared to older adults with back pain but without an LSS diagnosis [6]. LSS is also associated with a lower health-related quality of life compared to those in the general population [7, 8]. LSS places a large economic burden on our healthcare system as well. It is one the most common reasons for lumbar spine surgeries among older adults [9], and surgical procedures for degenerative lumbar spine conditions have been increasing for decades [10–13].

There are key gaps in our knowledge of LSS that make clinical decision-making challenging for patients and providers. One gap is that there are no standard diagnostic criteria for LSS, even though several recommendations exist [14, 15]. LSS has traditionally been viewed as a structural problem, and diagnosis often combines radiographic evidence from lumbar imaging tests and the characteristic clinical symptoms; however, there is no consensus that this is the best approach [1]. There is variability in how radiologists define and classify anatomic LSS [15–17], and although there are attempts to standardize anatomically defined LSS [18, 19] and some recent agreement on typical signs and symptoms associated with LSS [20–22], there is no “gold standard” for the clinical diagnosis of LSS. The use of radiologic findings for guiding care and decision-making is also problematic, since many individuals with anatomic LSS are asymptomatic [2, 23], and radiological findings of LSS have weak or no associations with symptoms and outcome [24–29]. Since optimal diagnostic criteria for LSS are uncertain [30], prognostic classification rather than diagnostic categories may be more useful for managing care and designing clinical trials [31]. Assessing multiple prognostic factors simultaneously may better reflect a person’s condition than a dichotomous diagnostic label for complex, multidimensional chronic conditions such as LSS [31, 32]. Improving our understanding of prognosis for older adults with LSS is needed to guide clinical decision-making for this growing public health issue.

Most patients with symptomatic LSS initially receive non-operative care before surgical consultation [14, 33–35]. However, there is a scarcity of data regarding the prognosis of LSS, what important subgroups exist, and what the key prognostic factors are for those seeking non-surgical treatment. This limitation has led to the recommendation for greater evidence on phenotyping the subgroups of LSS (i.e., defining and describing observable characteristics of subgroups) to better inform patients and providers regarding expected outcomes [14]. Better prognostic phenotyping can inform non-surgical treatment and may reduce unnecessary surgeries. However, there is limited evidence on prognosis for non-surgically treated patients presenting to surgical settings or other secondary care settings. Prospective studies on patients presenting to surgical settings suggest LSS symptoms are relatively stable or improve somewhat over time for many patients managed non-surgically [26, 35–38]. This information helps us to develop hypotheses concerning prognosis, potential subgroups, and important prognostic factors; but there is still little high-quality evidence to guide patient and clinician decision-making for patients pursuing non-surgical care.

This protocol describes the overall goal, aims, and methods of The Lumbar Stenosis Prognostic Subgroups for Personalizing Care and Treatment (PROSPECTS) Study. The overall goal of this project is to develop and evaluate a clinically useful model to predict long-term physical function of patients initiating non-surgical care for symptomatic LSS. To do this, we aim to identify clinically important phenotypes using prognostic trajectories and develop a well-performing predictive model to enable the creation of patient-centered, stratified care pathways for symptomatic LSS. The Specific Aims are 1) to identify clinically meaningful trajectories of long-term physical function and 2) to develop and evaluate a prognostic model to predict long-term physical function among adults ≥50 years initiating care for symptomatic LSS. Aim 1 has no specific hypothesis being tested, but we expect to identify multiple distinct trajectories defined by changes in physical function over 12 months. Our first hypothesis for Aim 2 is that chronicity; psychological factors such as depression, anxiety, and expectations; comorbidity burden; and the presence of particular comorbidities (e.g., lower-extremity osteoarthritis, widespread pain) will be important predictors of long-term physical function. Our second hypothesis for Aim 2 is that including these variables in a prognostic model will contribute to a well-performing and well-calibrated model to predict long-term patient-reported physical functional outcomes. The successful completion of this project will identify prognostic trajectories that can be easily applied to the clinic setting, and it will produce a cross-validated prognostic model for LSS. These findings can then be used to tailor treatment approaches for patient care and future clinical trials.

## Methods

### Study design

We are conducting an inception cohort study of adults 50 years and older who are initiating non-surgical care for symptomatic LSS in a secondary care setting. We recruit study participants from 2 sites, Duke spine clinics in Durham, North Carolina and UW Medicine spine clinics in Seattle, Washington. The University of Washington began recruiting participants in June of 2021, and Duke University started recruitment in August of 2021. Participants are followed for 12 months, with surveys completed at baseline, 3, 6, and 12 months. Ethics approval for this study was provided by the Institutional Review Boards at both the University of Washington (#STUDY00011262) and Duke University (#Pro00107101).

### Participants and eligibility criteria

We include patients ≥50 years old. We selected an age cut-off of 50 years because LSS is a condition that is associated with older age; we have used this criterion in prior studies of LSS [39, 40]. Participants must have symptomatic LSS, defined as patients reporting at least 3 of the following 4 symptoms: bilateral lower extremity symptoms, lower extremity pain greater than back pain, pain radiating into the buttock or lower extremity with standing or walking, and relief of lower extremity symptoms with sitting. These criteria have a high specificity (98%) for LSS among individuals ≥50 years old [21]. A new secondary care visit is defined as having no back-related visit or procedure in the prior 6 months [41], other than visits in primary care settings, which include physical therapy or chiropractic visits with a back-related diagnosis code. This is referred to as the index visit. We exclude patients with serious medical conditions that also have back pain as a symptom or limit walking such as inflammatory spondyloarthropathy, spinal malignancy, spinal infection, vascular claudication, developmental spine deformities, severe vascular disease that may limit ambulation, severe pulmonary disease that may limit ambulation, severe coronary artery disease that may limit ambulation, severe osteoporosis indicated by a vertebral compression fracture, and/or a history of cancer (excluding non-melanomatous skin cancer). A complete list of the exclusion criteria is listed in Table 1.

**Table 1:** Exclusion Criteria

Previous lumbar spine surgery	
Planning on surgical consult or seeking elective spine surgery within the next 6 months	
Back-related visit or procedure in prior 6 months (exception: Primary care settings)	
Planning on moving out of area or leaving Health System within the next 12 months	
Major medical problems or planned surgeries that would prevent you from being in the study for the next 12 months	
Inflammatory spondyloarthropathy	
Suspected spinal malignancy or infection at index visit	
Developmental spine deformities (e.g. spina bifida or tethered cord)	
Vascular claudication	
Severe vascular, pulmonary, or coronary artery disease which limits ambulation; including recent myocardial infarction (within 6 months)	
Trauma or joint replacement in past 12 month that continues to limit ambulation	
Severe osteoporosis as defined by at least one compression fractures	
History of cancer within past 5 years, excluding non-melanomatous skin cancer	
Severe cognitive impairment that would interfere with answering questions (assessed by Six-Item Cognitive Screening Questionnaire)	

### Recruitment

**Fig. 1:**
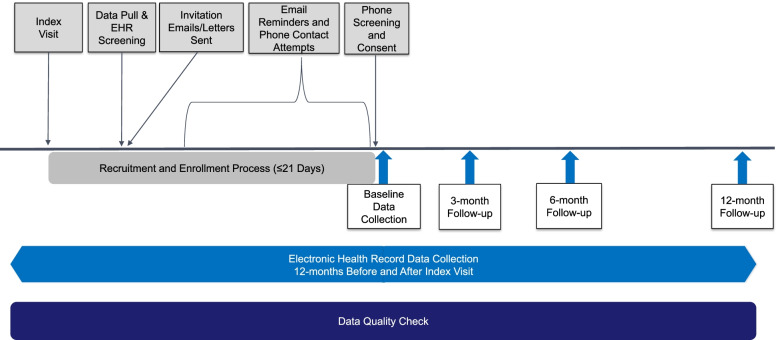
Schematic for the timing of recruitment and study procedures

**Fig. 2:**
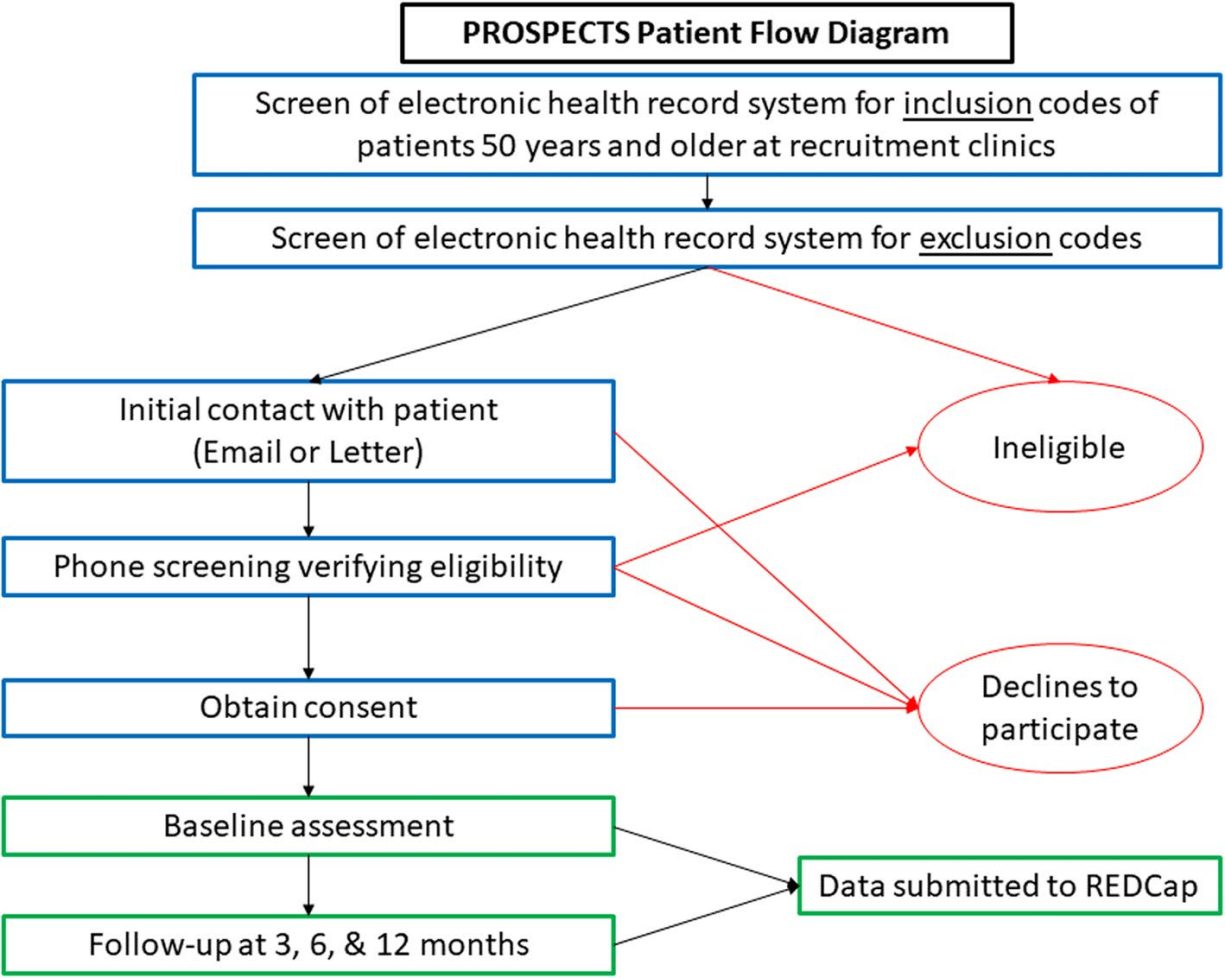
Flow chart of study screening, enrollment, and data collection

We are using weekly, automated data pulls from the electronic health record (EHR) at study sites to identify potential participants with an eligible index visit using queries for relevant International Classification of Diseases, Tenth Revision, Clinical Modification (ICD10) spine codes (Table 2). Because not all patients presenting with the clinical syndrome of LSS may be given a specific LSS diagnosis code during their initial visit, each week, we also search for patients with relevant ICD10 diagnosis codes that suggest possible LSS to identify potentially eligible participants. We then use the EHR to screen for exclusion diagnosis codes and prior visits with lumbar spine related diagnosis codes (Supplementary Table). Patients that meet these initial screening criteria are then approached by sending an email to all patients with an email address available in the medical record. Paper letters are mailed to those without an email address listed. The invitations explain the study, invite patients to join, and give them the option to opt-out of being contacted. Patients who do not opt-out within 4 days are then contacted by research staff to determine eligibility, obtain consent, and enroll participants. As this is an inception cohort study, the consent, enrollment, and the baseline survey completion must occur within 21 days of the index visit. Patients > 21 days from their index visit are no longer eligible for the study (Figs. 1 and 2). To conduct our screening processes, we obtained a waiver of consent and waiver of HIPAA authorization so the research staff will be able to access medical records to determine subject eligibility prior to approaching the subjects for study consent and HIPAA Authorization.

**Table 2:** Inclusion diagnosis codes for screening

ICD10 Code	Description
M48.00	Spinal stenosis, site unspecified
M48.05	Spinal stenosis, thoracolumbar region
M48.06x	Spinal stenosis, lumbar region
M48.07	Spinal stenosis, lumbosacral region
M48.08	Spinal stenosis, sacral and sacrococcygeal region
M54.4x	Lumbago with sciatica
M54.3x	Sciatica, unspecified site
M54.15	Radiculopathy, thoracolumbar region
M54.16	Radiculopathy, lumbar region
M54.17	Radiculopathy, lumbosacral region
M54.18	Radiculopathy, sacral and sacrococcygeal region
M51.3	Other thoracic, thoracolumbar and lumbosacral intervertebral disc degeneration
M51.35	Other intervertebral disc degeneration, thoracolumbar region
M51.36	Other intervertebral disc degeneration, lumbar region
M51.37	Other intervertebral disc degeneration, lumbosacral region
M51.05	Intervertebral disc disorders with myelopathy, thoracolumbar region
M51.06	Intervertebral disc disorders with myelopathy, lumbar region
M51.15	Intervertebral disc disorders with radiculopathy, thoracolumbar region
M51.16	Intervertebral disc disorders with radiculopathy, lumbar region
M51.17	Intervertebral disc disorders with radiculopathy, lumbosacral region
M47.15	Other spondylosis with myelopathy, thoracolumbar region
M47.16	Other spondylosis with myelopathy, lumbar region
M47.25	Other spondylosis with radiculopathy, thoracolumbar region
M47.26	Other spondylosis with radiculopathy, lumbar region
M47.27	Other spondylosis with radiculopathy, lumbosacral region
M47.28	Other spondylosis with radiculopathy, sacral and sacrococcygeal region
M43.00	Spondylosis, site unspecified
M43.05	Spondylolysis, thoracolumbar region
M43.06	Spondylolysis, lumbar region
M43.07	Spondylolysis, lumbosacral region
M43.08	Spondylolysis,sacral and sacrococcygeal region
M43.10	Spondylolisthesis, site unspecified
Q76.2	Congenital spondylolisthesis
M43.15	Spondylolisthesis, thoracolumbar region
M43.16	Spondylolisthesis, lumbar region
M43.17	Spondylolisthesis, lumbosacral region
M43.18	Spondylolisthesis, sacral and sacrococcygeal region

### Data collection

We collect data on the study participants using telephone interviews, web-based surveys, and queries of the EHR. At baseline, research staff complete screening for eligibility over the telephone. At that point, eligible patients can choose to complete the consent and enrollment process via telephone or by accessing a link to a REDCap survey. After obtaining informed consent, the baseline survey is completed via a web-based survey or telephone interview administered by research staff, depending on their preference. Participants are given the option of completing the 3, 6, and 12-month follow-up surveys via web-based survey or a telephone interview as well (Fig. 2). There is a 4-week window (1 week before and 3 weeks after the follow-up time point) to complete each follow-up assessment. For participants who do not respond to email and phone reminders to complete a follow-up assessment, research staff mails key outcome questionnaires (PROMIS Physical Function, pain intensity, and Global Rating of Change) to participants with a postage paid return envelope. Patients are offered a $20 reimbursement or equivalent gift for their time for each completed survey.

### Baseline measures

We use the NIH Task Force on Research Standards for Chronic Low Back Pain minimum dataset as a part of our baseline and outcome data collection [42]. We also use the Patient-Reported Outcomes Measurement Information System PROMIS 29 + 2 Profile v2.1 to collect many of our predictor and outcome variables [42, 43]. The PROMIS 29 includes 4-item short forms for physical function, anxiety, depression, fatigue, sleep disturbance, participation, and pain interference. Two additional short form cognition questions are added to create the PROMIS 29 + 2 Profile. Demographic data of age, sex, race, ethnicity, height, weight, education, education level, marital status, and employment are self-reported at baseline. Self-reported health characteristics of smoking history, falls history, duration of symptoms, and frequency of symptoms are collected during the baseline survey. We collect a self-reported checklist of co-occurring pain sites [42], and we measure comorbidity burden using the Functional Comorbidity Index. This is a comorbidity index that has been validated for functional limitation in a population with back pain [44, 45]. We ask participants about their expectation for recovery on an 11-point numerical rating scale (0 = no confidence in recovery, 10 = complete confidence in recovery) using the question: “How confident are you that your back and/or leg pain will be completely gone or much better 3 months from now?” We also include questions from the STarT Back screening tool [46]. Spine-related care and medication use before and after the index visit are obtained with self-report forms and from the EHR. Zip code, payor type, and type of index provider will be extracted from the EHR. We also abstract any available lumbar spine imaging findings from patients’ radiology reports in the medical record. All variables and their schedule for collection are presented in more detail in Table 3.

**Table 3:** Measures and schedule for data collection

Variable	Measure	Baseline	Follow-up
Descriptive Measures and Covariates			
Demographics	NIH CLBP dataset demographic questions (age, sex, race, ethnicity, height/weight, education, marital status)	**x**	
Smoking history	Categorical variable: current, former, or never smoker	**x**	
Impact of chronic pain	9 items on interference and function from PROMIS short form per NIH Taskforce minimum dataset [42]	**x**	
Sleep disturbance	PROMIS-29 Profile v2.1 short form	**x**	
Fatigue	PROMIS-29 Profile v2.1 short form		
Participation	PROMIS-29 Profile v2.1 short form		
Falls	Self-reported history of falls; prior injurious falls	**x**	
Imaging findings	Findings abstracted from diagnostic imaging reports	**x**	
Spine Treatments	Opioids, Medications, Injections, Physical Therapy, Psychological counseling, Surgery	**x**	**x**
Index Provider	Provider type (e.g. physician specialty, physician assistant)	**x**	
Candidate Predictors			
Demographics	age, sex, and body mass index	**x**	
Social determinants	Education, payer type	**x**	
Baseline Function	PROMIS SF v1.2 - Physical Function 8b	**x**	
Baseline Pain intensity (back and leg)	Average back or leg pain intensity past 7 days using numerical rating scale (Pain NRS)	**x**	
Chronicity	Duration and frequency of symptoms per NIH Taskforce Definition for chronic low back pain	**x**	
Expectations for Recovery	11-point numerical rating scale	**x**	
Depression and Anxiety	PROMIS-29 Profile v2.1 short forms	**x**	
Falls	Self-reported prior fall	**x**	
Prognostic Scale	STaRT Back Screening Tool	**x**	
Chronic Conditions	Functional Comorbidity Index^101^	**x**	
Comorbid Pain Conditions	Self-report checklist: stomach pain, upper and lower extremity joint pain, headaches, widespread pain [42].	**x**	
Outcomes			
Physical Function	PROMIS SF v1.2 - Physical Function 8b	**x**	**x**
Back-related disability	Oswestry Disability Index	**x**	**x**
Pain Interference	PROMIS-29 Profile v2.1 short form	**x**	**x**
Pain Intensity (back & leg)	Average back or leg pain intensity past 7 days using numerical rating scale (Pain NRS)	**x**	**x**
Global Rating of Change	5-point Likert scale of change in their condition (worse vs improved)		**x**
Spinal Stenosis symptoms	Swiss Spinal Stenosis Questionnaire (SSSQ)	**x**	**x**
Ability to Exercise	Patient Specific Functional Scale	**x**	**x**
Adverse Events	Falls, cauda equina, fractures, and infections		**x**

Candidate predictors variables were identified from these baseline variables. Predictors considered for model development were selected based on prior associations with chronic pain and back-related disability. We have also selected candidate predictors that are commonly found in EHRs or are typically included as a part of clinical practice. Candidate predictors are denoted in Table 3.

### Outcomes

All outcome measures are collected at baseline and 3, 6, and 12 months after the index visit. The primary outcome is the 8-item PROMIS Physical Function (PF) Short Form, which measures self-reported mobility and physical function. It is a generic measure, rather than a disease-specific measure. Each item is scored from 1 (unable to do) to 5 (not limited at all or without difficulty). The raw score is converted into a standardized T-score, where 50 represents the population mean with a standard deviation of 10 [48]. We chose PROMIS PF because of its ability to generalize the finding across a wide variety of conditions. The PROMIS Pain Interference 4-item Short Form is a secondary outcome. Additional secondary outcomes include the Oswestry Disability Index (ODI). The ODI is a 10-question back-related disability questionnaire that asks about activities commonly affected by low back pain. It is scored from 0 (no disability) to 100 (maximal disability). It has been shown to have validity, good responsiveness, and acceptable reliability in patients with spine conditions and spinal stenosis [49–52]. Average back pain and leg pain intensity over the past 7 days is measured using a 0–10 numerical rating scale (NRS) [53]. We include the Swiss Spinal Stenosis Questionnaire (SSSQ), which is a self-report measure on the symptom severity and functional disability related to spinal stenosis. The SSSQ consists of 3 subscales: severity of symptoms, physical function, and satisfaction after treatment. We measure the severity of symptoms and function subscales at all time points and include one question on how satisfied participants are with their overall result of any treatments at 3, 6, and 12 months. The symptom scale is scored from 1 to 5 and the function and the satisfaction scales from 1 to 4, with higher total scores representing worse outcomes [47, 54–56]. We measure self-rated ability to exercise using the Patient Specific Functional Scale, which asks patients to rate their ability to complete an activity (exercise) on an 11-point scale (0 = unable to perform and 10 = able to perform fully) and has been validated in patients with LSS [57, 58]. Lastly, we are collecting Global Rating of Change since baseline using a 7-point Likert scale. All secondary outcome measures are described in further detail in Table 3.

### Analysis

#### Aim 1

First, we will describe the overall prognosis by estimating the average outcome scores of the cohort at baseline and then 3, 6, and 12 months using the mean and standard deviation for all outcome measures. We will also describe mean outcomes stratified by key baseline characteristics such as sex and comorbid health conditions, and we will estimate the proportion of patients experiencing a clinically important improvement in function (> 2 points for PROMIS PF; ≥10 points for ODI). To provide better context for these outcomes we will also describe the type and amount of spine-related treatments and procedures patients receive.

Next, we will identify distinct phenotypes using PROMIS PF T-scores at baseline and 3, 6, and 12 months using group-based trajectory modeling, a useful method to model dynamic outcomes over time [59, 60]. Each individual will be assigned to one trajectory group based on the probabilities of individual membership in each physical function trajectory group generated from the model. We will estimate models with 2 or more trajectories using an iterative process, where models differing by one class are successively run and compared using goodness of fit measures. Final model selection is based on the ability of the model to discriminate between trajectories, clinical interpretability and meaningfulness, and having a reasonable sample size in each trajectory (> 5% of total sample) [61]. We will also identify trajectories for secondary outcome measures. After identifying trajectory groups, we will characterize patients within the trajectory groups using baseline variables.

#### Aim 2

In Aim 2 we will develop and evaluate the performance of a multivariable prognostic model to predict 12-month physical function. First, we will describe unadjusted associations for each pre-selected candidate predictor and investigate non-linear associations for continuous predictor variables. We will include all our candidate predictors in the initial model. We will determine final predictors in the model using a penalized regression method, the least absolute shrinkage and selection operator (LASSO). LASSO is a supervised method for variable selection and shrinkage. It produces a parsimonious model without overfitting the data, and it avoids bias from variable selection based on statistical significance and traditional stepwise selection procedures [62, 63]. The optimal model will be determined based on performance, parsimony, and perceived utility in clinical settings. Additional analysis will evaluate other machine learning methods, such as elastic net, random forests, and ensemble methods such as super learner [64–66] to assess the robustness of the initial model and predictors included.

We will conduct internal validation to examine model performance. Internal validation for the performance of the selected model will be tested using cross-validation procedures such as k-folds. We will assess performance of the prognostic model by evaluating its calibration, goodness of fit, and prediction error, key properties of prognostic models [63, 67]. We will test calibration (agreement between observed and predicted outcomes) using calibration plots. The goodness of fit for each model will be evaluated using the R^2^ and Akaike information criterion. Prediction error will be assessed with root mean squared error, which estimates the spread of the residuals around the predicted outcome and can be interpreted as the standard deviation of the residuals. We will account for missing data by using appropriate methods such as multiple imputation or inverse-probability weighting for missing outcomes due to attrition. Results will be reported following the Transparent Reporting of a multivariable prediction model for Individual Prognosis Or Diagnosis (TRIPOD) statement and checklist [68], or the Strengthening the Reporting of Observational Studies in Epidemiology (STROBE) statement guidelines as appropriate [69].

### Sample size estimate

We used criteria recently recommended by Riley et al. to estimate the sample size required to develop a multivariable, prognostic model using a continuous outcome measure (e.g., PROMIS Physical Function t-score) [70]. We estimated the required sample size using the corresponding Stata package *pmsampsize*. We selected an expected R^2^ of 0.40 based on the R^2^ values of 2 back-related prediction models from recent studies [71, 72]. Based on the number of candidate predictors selected, we anticipated up to 25 predictor parameters. We assumed a mean PROMIS PF T-score of 35 and standard deviation of 6 for patients with LSS [50]. Using these estimates and their recommended level of shrinkage (0.90), a minimal sample size of 400 is required to develop this multivariable prognostic model. Suggestions for sample size when using group-based trajectory modeling are less clear, but key properties of the maximum likelihood function underlying group-based trajectory modeling are reliable in sample sizes as small as 500 [73]. Thus, to accomplish both aims, the required sample size is 500 participants. We plan to recruit up to 625 participants to ensure we’ll reach our target sample size of 500, with an attrition rate of up to 20%.

## Discussion

The PROSPECTS study will be one of the largest cohorts of individuals with symptomatic LSS initiating new episodes of non-surgical care. Consequently, this study will provide some of the highest level of evidence on the prognosis of LSS in non-surgical settings to date. Little evidence exists to guide patients and clinicians in making decisions regarding self-management, non-surgical care pathways, and when to consider surgery for this population. There is a critical need for better prognostic information on LSS-related pain and disability to guide patient decision-making and for researchers designing trials to compare treatments. To improve our understanding of prognosis for LSS, this study will identify prognostic phenotypes based on trajectories of long-term function, and the successful completion of this project will produce a cross-validated prognostic model for LSS that can be used to tailor treatment approaches for patient care and clinical trials.

There are several strengths of this project. First, we are using the optimal study design for prognostic research, an inception cohort study [74]. This design enrolls people at a uniform, salient event, such as a new healthcare visit, and then prospectively follows them over time. Thus, it allows researchers to observe eventual patient outcomes from this common timepoint [74]. Second, many studies that develop prognostic models use smaller samples and do not generalize well [75], but we used recent recommendations to determine a sufficient sample size when developing a multivariable, prognostic model using a continuous outcome measure to avoid overfitting and ensure precise estimates [70]. Third, we are using 2 separate health systems and sites, which further strengthens the generalizability of our results. Fourth, we are leveraging EHRs to facilitate recruitment by identifying most potentially eligible patients at our 2 study sites. This can help reduce selection bias by attempting to generate a sample that more closely reflects the population to which the results will be applied. Lastly, we will create a model that can be easily automated into clinical decision support tools by using predictors that are commonly found in EHRs or are typically included as a part of clinical practice. This should facilitate integration with electronic data collection methods.

### Anticipated challenges and contingency plans

One challenge all studies commonly encounter is meeting recruitment goals and retaining participants once they are enrolled. If we do not meet our recruitment benchmarks, we will consider extending duration of recruitment or searching patient lists from additional clinics at the study sites. For example, we may consider expanding our screening processes to physical therapy clinics. The lack of an eligibility criterion on imaging-confirmed LSS may be viewed as a limitation by some, but we chose to use only clinical criteria for symptomatic LSS for a couple of reasons. As mentioned previously, radiologic findings are poorly associated with symptoms and disability [24–29], and since optimal diagnostic criteria for LSS is uncertain, prognostic classification rather than diagnostic categories may be more useful for managing care and designing clinical trials [31]. Additionally, we thought it was less feasible and potentially inefficient to create a prognostic model that would require patients to have diagnostic imaging to determine if the prognostic model could be applied to them or not. Using solely clinical criteria provides greater flexibility for how an eventual prognostic model can be applied. However, we are collecting and abstracting the radiology reports for all participants that have these data available in their EHR, and these results will help characterize the LSS radiologic findings present in this cohort and can be used in sensitivity analyses. Lastly, external validation of the eventual PROSPECTS prognostic model will be required before it can be confidently applied in clinics. Recruiting a second cohort for external validation is beyond the scope of this project, but it is an important priority for future research. This is the next step that will move us towards our long-term goal of creating better patient-centered and higher-value approaches to care for individuals with symptomatic LSS.

## Supplementary Information

**Additional file 1: Supplementary Table.** Exclusion diagnosis codes for screening**.**

## Data Availability

Not applicable.

## Abbreviations

LSS: Lumbar Spinal Stenosis
PROSPECTS: Prognostic Subgroups for Personalizing Care and Treatment Study
ICD10: International Classification of Diseases, Tenth Revision, Clinical Modification
EHR: Electronic Health Record
HIPAA: The Health Insurance Portability and Accountability Act
PROMIS: Patient Reported Outcomes Measurement Information System
PF: Physical Function
ODI: Oswestry Disability Index
NRS: Numerical Rating Scale
SSSQ: Swiss Spinal Stenosis Questionnaire
LASSO: Least Absolute Shrinkage and Selection Operator
TRIPOD: Transparent Reporting of a multivariable prediction model for Individual Prognosis Or Diagnosis
