# Supplementary material for: The Lumbar Stenosis Prognostic Subgroups for Personalizing Care and Treatment (PROSPECTS) study: protocol for an inception cohort study
Source: BMC Musculoskelet Disord. 2022 Jul 21;23:692. doi: 10.1186/s12891-022-05598-x (PMC9306038; doi:10.1186/s12891-022-05598-x)
Supplement: Supplementary file 1 — Additional file 1: Supplementary Table. Exclusion diagnosis codes for screening. [file 12891_2022_5598_MOESM1_ESM.docx]

**Supplementary Table. Exclusion diagnosis codes for screening**

| **ICD10 Code** | **Exclusion Category**  **(and search timeframe)** |
| --- | --- |
| S33.x Dislocation and sprain of joints and ligaments of lumbar spine and pelvis  S39.002 Unspecified injury of muscle, fascia and tendon of lower back  S39.012 Strain of muscle, fascia and tendon of lower back  S39.092 Other injury of muscle, fascia and tendon of lower back  S39.92 Unspecified injury of lower back  M43.x Other deforming dorsopathies  M47.x Spondylosis  M48.x Other spondylopathies  M49.x Spondylopathies in diseases classified elsewhere  M51.x Thoracic, thoracolumbar, and lumbosacral intervertebral disc disorders  M53.2X5 Spinal instabilities, thoracolumbar region  M53.2X6 Spinal instabilities, lumbar region  M53.2X7 Spinal instabilities, lumbosacral region  M53.2X8 Spinal instabilities, sacral and sacrococcygeal region  M53.3 Sacrococcygeal disorders, not elsewhere classified  M53.80 Other specified dorsopathies, site unspecified  M53.85 Other specified dorsopathies, thoracolumbar region  M53.86 Other specified dorsopathies, lumbar region  M53.87 Other specified dorsopathies, lumbosacral region  M53.88 Other specified dorsopathies, sacral and sacrococcygeal region  M53.9 Dorsopathy, unspecified  M54.15 Radiculopathy, thoracolumbar region  M54.16 Radiculopathy, lumbar region  M54.17 Radiculopathy, lumbosacral region  M54.18 Radiculopathy, sacral and sacrococcygeal region  M54.3x Sciatica  M54.4x Lumbago with sciatica  M54.5 Low back pain  M54.89 Other dorsalgia  M54.9 Dorsalgia, unspecified  M62.830 Muscle spasm of back  M99.03 Segmental and somatic dysfunction of lumbar region  M99.04 Segmental and somatic dysfunction of sacral region | Back-related visit or procedure in prior 6 months.  Comment: IF one of these lumbar codes is present within the 6 months prior to the identified/index visit AND that prior visit is at one of our identified spine clinics THEN exclude. |
| M45.x Ankylosing spondylitis  M00.88 Arthritis due to other bacteria, vertebrae  M02.38 Reiter's disease, vertebrae  M02.88 Other reactive arthropathies, vertebrae | Inflammatory spondyloarthropathy  (within prior 12 months) |
| C41.2 Malignant neoplasm of vertebral column  C41.4 Malignant neoplasm of pelvic bones, sacrum and coccyx  C70.1 Malignant neoplasm of spinal meninges  C72.0 Malignant neoplasm of spinal cord  C72.1 Malignant neoplasm of cauda equina  D17.348 Neoplasm of uncertain behavior of spinal cord  M46. x Other inflammatory spondylopathies  M87.x Osteonecrosis  A18.01 Tuberculosis of spine  M89.106 Tuberculosis of spine  D17.2996 Intraspinal abscess and granuloma  D17.2997 Extradural and subdural abscess, unspecified  M00.08 Staphylococcal arthritis, vertebrae  M00.18 Pneumococcal arthritis, vertebrae  M00.28 Other streptococcal arthritis, vertebrae  M01.X8 Direct infection of vertebrae in infectious and parasitic diseases classified elsewhere  G83.4 Cauda equina syndrome  G95.81 Conus medullaris syndrome | Suspected spinal malignancy or infection or Cauda Equina Syndrome  (within prior 12 months) |
| I73.9 Peripheral vascular disease, unspecified | Vascular claudication  (within prior 12 months) |
| Q05.x Spina bifida  Q06.x Congenital malformation of spinal cord  Q67.5 Congenital deformity of spine | Developmental spine deformities  (within prior 12 months) |
| I60.x Nontraumatic subarachnoid hemorrhage  I61.x Nontraumatic intracerebral hemorrhage  I62.x Other and unspecified nontraumatic intracranial hemorrhage  I63.x Cerebral infarction  I71.x Aortic aneurysm and dissection  I72.2-I72.9 Other aneurysm  I74.x Arterial embolism and thrombosis  I82.x Other venous embolism and thrombosis | Severe vascular disease which limits ambulation (new within 6 months) |
| I26.x Pulmonary embolism  J85-J86 Suppurative and necrotic conditions of the lower respiratory tract  J90-J94 Other diseases of the pleura | Severe pulmonary disease which limits ambulation  (new within 6 months) |
| I21.x Acute myocardial infarction  I22.x Subsequent ST elevation (STEMI) and non-ST elevation (NSTEMI) myocardial infarction  I23.x Certain current complications following ST elevation (STEMI) and non- ST elevation (NSTEMI) myocardialinfarction (within 28 days)  I24.x Other acute ischemic heart diseases | Severe coronary artery disease which limits ambulation including recent myocardial infarction  (new within 6 months) |
| M80.08 Age-related osteoporosis with current pathological fracture, vertebra(e)  M80.88 Other osteoporosis with current pathological fracture, vertebra(e)  M48.5x Collapsed vertebra, not elsewhere classified  S22.000x Wedge compression fracture of unspecified thoracic vertebra  S22.0x0 Wedge compression fracture of x thoracic vertebra  S32.000 Wedge compression fracture of unspecified lumbar vertebra  S32.0x0 Wedge compression fracture of x lumbar vertebra | Severe osteoporosis indicated by compression fracture  (new within 6 months) |
| C00-C14 Malignant neoplasms of lip, oral cavity and pharynx  C15-C26 Malignant neoplasms of digestive organs  C30-C39 Malignant neoplasms of respiratory and intrathoracic organs  C40-C41 Malignant neoplasms of bone and articular cartilage  C43-C44 Melanoma and other malignant neoplasms of skin  C45-C49 Malignant neoplasms of mesothelial and soft tissue  C50 Malignant neoplasms of breast  C51-C58 Malignant neoplasms of female genital organs  C60-C63 Malignant neoplasms of male genital organs  C64-C68 Malignant neoplasms of urinary tract  C69-C72 Malignant neoplasms of eye, brain and other parts of central nervous system  C73-C75 Malignant neoplasms of thyroid and other endocrine glands  C7A Malignant neuroendocrine tumors  C7B Secondary neuroendocrine tumors  C76-C80 Malignant neoplasms of ill-defined, other secondary and unspecified sites  C81-C96 Malignant neoplasms of lymphoid, hematopoietic and related tissue  D00-D09 In situ neoplasms  D37-D48 Neoplasms of uncertain behavior, polycythemia vera and myelodysplastic syndromes  D49 Neoplasms of unspecified behavior | History of cancer excluding non-melanomatous skin cancer  (within prior 12 months) |
| F01.x Vascular dementia  F02.x Dementia in other diseases classified elsewhere  F03.x Unspecified dementia  G30.x Alzheimer's disease  G31.09 Other frontotemporal dementia  G31.83 Dementia with Lewy bodies | Severe cognitive impairment (e.g. Dementia)  (within prior 12 months) |
